# Neurovascular Disorders: Novel Perspectives on Pathogenesis, Diagnosis, and Treatment

**DOI:** 10.1155/2016/9291327

**Published:** 2016-01-18

**Authors:** David Hasan, Nohra Chalouhi, Aaron S. Dumont, Robert M. Starke, Pascal Jabbour

**Affiliations:** ^1^University of Iowa, Iowa City, IA, USA; ^2^Thomas Jefferson University, Philadelphia, PA, USA; ^3^Tulane University, New Orleans, LA, USA; ^4^University of Virginia, Charlottesville, VA, USA

Neurovascular disorders include subarachnoid hemorrhage, intracranial aneurysms, arteriovenous malformations, cavernous malformations, carotid disease, acute ischemic stroke, intracerebral hemorrhage, and moyamoya disease. Recently, there has been a greater understanding of both the genetic links and the basic mechanisms behind the pathophysiology of neurovascular diseases. Additionally, there have been significant advances in diagnosis, medical treatment, and microsurgical/endovascular therapies.

This special issue on neurovascular disorders sheds light on several important aspects of common neurovascular disorders including acute ischemic stroke, arteriovenous malformations (AVMs), and subarachnoid hemorrhage.

Among several nicely conducted studies published in this issue, Y. Zhang et al. provide us with a study demonstrating that somatosensory and brainstem auditory evoked potentials assessed between 4 and 7 days after severe stroke onset may predict unfavorable outcome. S. Hillman et al. study temporal changes in the quality of acute stroke care in five national audits in Europe and report a general trend towards a better quality of stroke care over time signaling that monitoring of stroke care performance contributes to the improvement of quality of care. In a nicely designed experiment, N. Iwata et al. report that repeated administration of etanercept may prevent exacerbation of cerebral ischemic injury in diabetic rats. W. Li et al. demonstrate that* SAMHD1* gene mutations are associated with cerebral large-artery atherosclerosis. In a pilot study, C.-P. Chung et al. report that the level of circulating endothelial progenitor cell is associated with cerebral vasoreactivity. With regard to AVMs, M. Xu et al. provide us with a timely review of animal models for studying cerebral AVMs. Finally, A. Benet et al. elegantly demonstrate the feasibility of implanting a 3D-printed brain aneurysm model in human cadavers for use in neurosurgical research, case planning, and operative training.

Improvements in diagnosis, imaging, and therapies will lead to improved outcomes for neurovascular disorders. It is crucial to study the safety and efficacy of new therapies and compare them to the existing modalities to identify the best options for our patients. Within this compilation of studies, we hope to elucidate areas of uncertainty, recent developments, and clinical necessity.

*David Hasan* *Nohra Chalouhi* *Aaron S. Dumont* *Robert M. Starke* *Pascal Jabbour*

